# Retrospective Evaluation of Clinical Bleeding in Dogs With Anticoagulant Rodenticide Toxicity—A Multi-Center Evaluation of 62 Cases (2010–2020)

**DOI:** 10.3389/fvets.2022.879179

**Published:** 2022-05-23

**Authors:** Sarah Stroope, Rebecca Walton, Jonathan Paul Mochel, Lingnan Yuan, Brittany Enders

**Affiliations:** ^1^Department of Veterinary Clinical Sciences, College of Veterinary Medicine, Iowa State University, Ames, IA, United States; ^2^Department of Biomedical Sciences, College of Veterinary Medicine, Iowa State University, Ames, IA, United States; ^3^College of Veterinary Medicine, North Carolina State University, Raleigh, NC, United States

**Keywords:** anticoagulant rodenticide, cavitary hemorrhage, hemostasis, mucosal hemorrhage, transfusion

## Abstract

**Objective:**

To evaluate the most common locations of hemorrhage in dogs diagnosed with anticoagulant rodenticide intoxication.

**Animals:**

Dogs presenting with hemorrhage secondary to anticoagulant rodenticide intoxication between at two university veterinary teaching hospitals.

**Procedures:**

Medical records were searched from the years 2010 through 2020 and all records from dogs treated for hemorrhage secondary to anticoagulant rodenticide intoxication were reviewed. Dogs were diagnosed with anticoagulant rodenticide intoxication based on the combination of known exposure and prolonged coagulation testing, including prothrombin and activated thromboplastin time, or based on gas chromatography-mass spectrometry (GCMS). The diagnosis of hemorrhage was made based on physical exam findings, point-of-care ultrasound findings or radiography.

**Results:**

Sixty-two dogs met the inclusion criteria and were included in the study. The most common sites of hemorrhage included: pleural space (hemothorax 37%), pulmonary parenchyma (24%), abdomen (24%), skin/subcutaneous (21%), gastrointestinal tract (18%), pericardium (13%), oral cavity (13%), nasal cavity (11%), ocular (8%), and urinary tract (7%). Overall, forty-five dogs (73%) had evidence of cutaneous or mucosal hemorrhage while thirty-three (53%) of dogs had evidence of cavitary hemorrhage. Forty-five percent of dogs had hemorrhage noted at only one site, while 55% experienced hemorrhage at more than one site. The location of hemorrhage and total number of hemorrhagic sites was not associated with survival or transfusion requirement.

**Conclusions and Clinical Relevance:**

In conclusion, this study highlights that dogs with anticoagulant rodenticide intoxication present with diverse locations of hemorrhage and the majority of dogs had non-cavitary hemorrhage noted.

## Introduction

Rodenticide ingestion has been among the top ten most common toxicities of dogs for the last two decades according to annual reports conducted by the American Society for Prevention of Animal Cruelty (ASPCA) (1–3). Despite legislation passed in the United States in 2011 banning the use of second-generation anticoagulant rodenticides from consumer use, anticoagulant rodenticide intoxication remains a common toxicity reported in domestic animals (1–3). Warfarin, the first anticoagulant rodenticide, was registered for use in 1950 and was widely used for rodent control (4). The development of resistance to warfarin in the following decades led to the development of second-generation anticoagulant rodenticides such as brodifacoum, bromadiolone, and diphacinone. These second-generation anticoagulants are potent intoxicants and have a significantly longer half-life than first-generation anticoagulants (1). These characteristics result in greater risk of severe morbidity and mortality to dogs that are exposed to these rodenticides.

Anticoagulant rodenticides prevent the recycling of vitamin K, which is required to activate coagulation factors II, VII, IX, and X via γ-carboxylation (5, 6). For vitamin K dependent coagulation factors to become functional, reduced vitamin K, hydroquinone, is required for posttranslational γ-carboxylation. During the γ-carboxylation process, hydroquinone is oxidized into inactive vitamin K epoxide. The enzyme vitamin K epoxide reductase is required to catalyze the conversion of inactive vitamin K epoxide back into hydroquinone (6). Anticoagulant rodenticides act to antagonize the action of vitamin K epoxide reductase, and consequently hydroquinone is no longer available to activate vitamin dependent coagulation factors (6). The body's supply of active coagulation factors II, VII, IX, and X become depleted and result in significant hemorrhage after 3–5 days (6). Patients often present to veterinary hospitals with non-specific complaints such as lethargy, anorexia, and pallor (5–7). Hemorrhage secondary to anticoagulant rodenticide may occur at any site and has been reported to cause body cavity hemorrhage, gastric hemorrhage, uterine hemorrhage, hemorrhage into the upper airways, pericardium, joints and eyes (8–16). Despite the various reports regarding potential sites of hemorrhage there is no published literature that identifies the incidence of locations of hemorrhage in dogs with anticoagulant rodenticide intoxication. As clinical signs overlap with those caused by coagulopathies of varying etiologies, the establishment of common locations of hemorrhage may allow clinicians to more quickly and accurately diagnose patients with anticoagulant rodenticide intoxication when history of ingestion is unknown. The goal of this study was to determine the most frequent sites of hemorrhage associated with anticoagulant rodenticide intoxication in dogs. Secondary objectives of this study were to identify the incidence of single vs. multiple sites of hemorrhage and to determine if there was any correlation between location and number of sites of hemorrhage with transfusion requirement or patient outcome.

## Materials and Methods

### Case Selection Criteria and Medical Records Review

Medical records from the XXX and XXX from the years 2010 through 2020 were searched for all dogs treated for hemorrhage secondary to anticoagulant rodenticide ingestion. Dogs were included in the study when anticoagulant ingestion was confirmed based on history, including known access or witnessed ingestion in combination with prolonged prothrombin time or with gas chromatography mass spectroscopy (GCMS). Dogs without definitive diagnosis of anticoagulant intoxication based on historical questions or laboratory data were excluded and if dogs were suspected and treated for rodenticide toxicity but no known exposure was noted they were excluded from data collection. Diagnosis of hemorrhagic sites were made based on either direct visualization of hemorrhage from nasal cavity, skin, eyes or oral cavity, or findings on diagnostic imaging including radiographs or POCUS in combination with diagnostic paracentesis (peritoneum/retroperitoneum, pleural space, and pericardium). Gastrointestinal hemorrhage was diagnosed based on the presence of hematemesis, melena or hematochezia, urinary tract hemorrhage was based on presence of hematuria and suspected pulmonary hemorrhage diagnosis was based on evidence of radiographic changes consistent with pulmonary infiltrates or hemoptysis.

All dogs were clinically managed according to clinician discretion and all dogs received treatment with Vitamin K. Decision for transfusions were made on clinician discretion and client consent. Due to the retrospective nature of this study informed client consent was not obtained at the time of data collection.

### Data Analysis

Statistical analyses were performed using commercially available software (R version 4.0.3, R Foundation for Statistical Computing, Vienna, Austria). The relationship between survival rate and the number of bleeding locations was evaluated by logistic regression. To assess the effect of each bleeding site on the survival rate, univariate logistic regressions were performed. Similar analyses were performed for the relationship between transfusion and the number of bleeding sites. These effects were considered statistically significant for *P*-values < 0.05.

## Results

Seventy-three records were reviewed and sixty-two dogs met the inclusion criteria based on prolongation of prothrombin time (PT) in combination with known exposure or witnessed ingestion or based on GCMS testing. Five dogs were diagnosed based on GCMS while the remaining fifty-seven dogs were diagnosed based on the combination of known access/exposure or witnessed ingestion and prolongation of clotting times. Each patient's presenting complaint and location of hemorrhage were recorded including number of sites of hemorrhage. Other variables collected included: blood products administered and survival to discharge.

**Figure 1:**
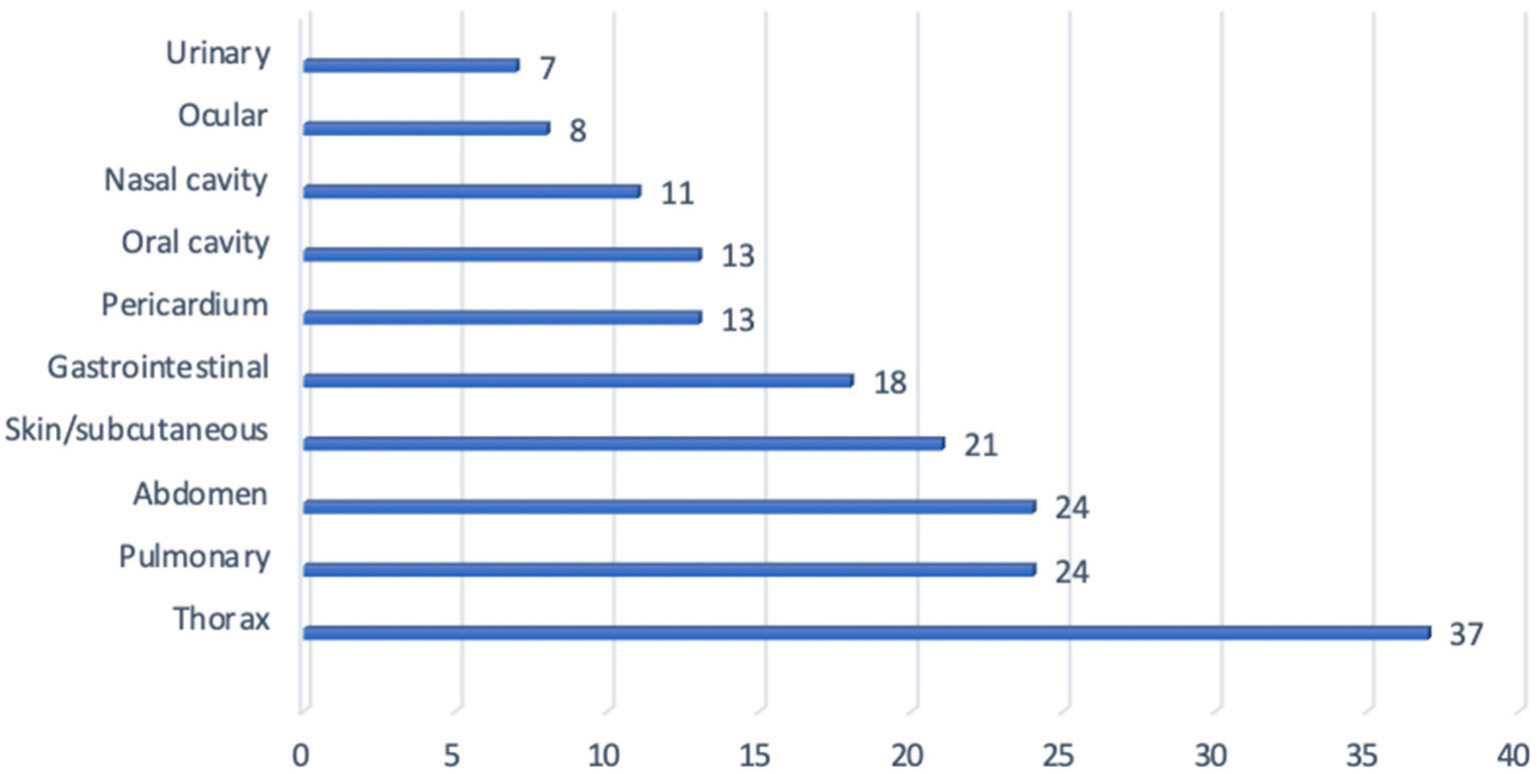
Locations of hemorrhage.

**Figure 2:**
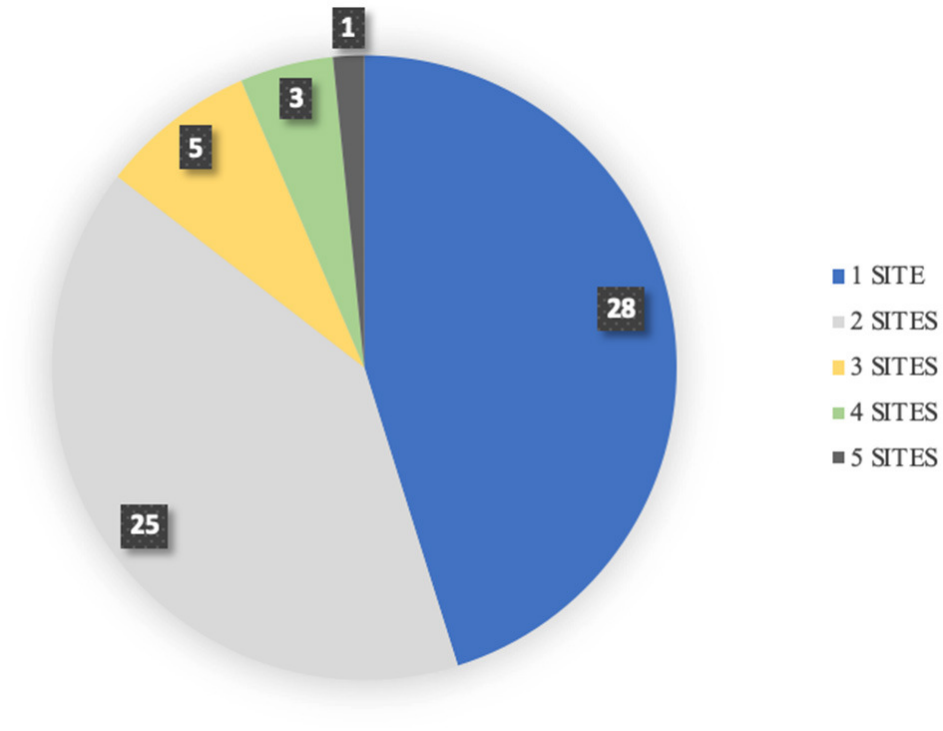
Sites of hemorrhage.

Sites of reported hemorrhage included: pleural space, pulmonary parenchyma, abdomen, skin, gastrointestinal tract, pericardial space, oral, nasal, ocular and urinary tract. The most common sites of hemorrhage included: pleural space (hemothorax 37%), pulmonary parenchyma (24%), abdomen (24%) [hemoabdomen (23%) and retroperitoneal space (2%)], skin/subcutaneous (21%), gastrointestinal tract (18%), pericardium (13%), oral cavity (13%), nasal cavity (11%), ocular (8%), and urinary tract (7%) (Figure 1). Hemoarthrosis was not reported in any dog. When assessing the overall results, forty-five dogs (73%) had evidence of cutaneous or mucosal hemorrhage while thirty-three (53%) dogs had evidence of cavitary hemorrhage. Total sites of hemorrhage in this study were 109 sites. Cavitary hemorrhage including: hemothorax, hemoabdomen or pericardial effusion accounted for 44 of the sites of hemorrhage (40%). Non-cavitary hemorrhage accounted for 65 sites (60%) and was present in 73% of dogs presenting for anti-coagulant rodenticide intoxication. Twenty-eight dogs (45%) had hemorrhage noted at 1 site and 25 dogs (40%) had hemorrhage reported at two sites. Dogs with multiple locations of hemorrhage included five dogs (8%) with three sites, three dogs (5%) with four sites, and one dog (2%) with five sites of hemorrhage (Figure 2).

Dogs presenting with anticoagulant rodenticide often present with hemorrhage at more than one location (5). Twenty-eight (82%) of dogs with hemorrhage at multiple sites had a combination of cavitary and non-cavitary hemorrhage. Three dogs (9%) had only cutaneous or mucosal hemorrhage and three dogs (9%) had only cavitary hemorrhage. The most common combination of hemorrhagic sites included pleural effusion (hemothorax) and pulmonary hemorrhage in 12% of dogs. Fifty-four dogs (87%) survived to discharge. Four dogs died and four were euthanized. Of the four dogs that died three had necropsy exams perform that revealed severe, diffuse hemorrhage include intra-cranial hemorrhage and retroperitoneal hemorrhage in one dog and the other two dogs had severe hemopericardium, hemothorax and hemoabdomens noted. There was no correlation found between survival and the location of hemorrhage or with the number of sites of hemorrhage (*p* = 0.69).

Forty-eight dogs (78%) received transfusion with blood products. The need for transfusion was based on clinician discretion. Fourteen dogs did not receive a transfusion. Of the fourteen dogs that did not receive a transfusion, six did not survive to discharge. The remaining eight cases that survived to discharge but did not receive a transfusion, five did not receive a transfusion due to the mild nature of hemorrhage and three did not receive a transfusion due to owner finances. All dogs received Vitamin K therapy. Of the dogs that did receive a transfusion, 46 (96%) were transfused with canine fresh frozen plasma. Two dogs (4%) received canine frozen plasma. Twenty-four dogs (50%) received a transfusion with canine packed red blood cells and one dog (2%) received canine whole blood. Four dogs (8%) received an autotransfusion. The location of hemorrhage was not associated with the need for any blood product (*p* = 0.95). Dogs were hospitalized for an average of 39 h (range 1–144 h).

## Discussion

Anticoagulant rodenticide is a common toxicity reported in dogs and is associated with clinical hemorrhage due to vitamin K antagonism. Vitamin K antagonism results in depletion of vitamin K dependent coagulation factors, II, VII, IX and X (5). The clinical signs of anticoagulant rodenticide intoxication vary depending on the site of hemorrhage and can include non-specific signs such as dyspnea, coughing/hemoptysis, lethargy, inappetence, vomiting, respiratory distress and cough (5, 9, 14). Dogs also present with a variety of clinical exam findings including shock, tachycardia, weak pulses, pallor, increased respiratory rate and effort, subcutaneous hematomas or ecchymosis and it is essential to differentiate anticoagulant rodenticide intoxication from other coagulopathies (5, 7). Diagnosis of anticoagulant rodenticide is often made based on the combination of exposure, clinical signs and prolonged coagulation times (5). Additional diagnostics utilized in the diagnosis of anticoagulant rodenticide intoxication includes high-performance liquid chromatography or GCMS (7). While it is documented that toxicities associated with anticoagulant rodenticide intoxication may cause hemorrhage at any site within the body, it is classically associated with cavitary hemorrhage (14, 17, 18). Previous reports describe high percentages of thoracic and abdominal radiographic abnormalities, 82 and 71%, respectively, however, specific locations of hemorrhage have not been previously evaluated (5). In addition to the more commonly noted cavitary hemorrhage, multiple case reports have described unusual locations of hemorrhage including: upper airways, gastric lumen and uterus (12–14). Due to the varying, non-specific presenting signs associated with anticoagulant rodenticide intoxication, the goal of this retrospective analysis was to evaluate the most common locations of hemorrhage in dogs diagnosed with anticoagulant rodenticide intoxication.

In this study, hemorrhage occurred at multiple sites including: thorax, abdomen, skin/subcutaneous tissue, gastrointestinal tract, oral cavity, nasal cavity, eyes and urinary tract. Hemorrhagic pleural effusion, with a diagnosis of hemothorax, was the most prevalent single site of hemorrhage (37%), however, when assessing the overall results, 73% of dogs had evidence of cutaneous or mucosal hemorrhage in contrast to 53% of dogs with evidence of cavitary hemorrhage. While cutaneous and mucosal hemorrhage is often considered to be associated with a defect in primary hemostasis (thrombocytopenia or thrombocytopathia), cutaneous and mucosal hemorrhage may be secondary to defects in secondary hemostasis, including anticoagulant rodenticide intoxication (19). In this study, non-cavitary hemorrhage was the most predominant category of hemorrhage in regards to overall sites of hemorrhage, 60% and also present in 73% of cases presenting for anticoagulant rodenticide intoxication.

Due to the nature of anticoagulant rodenticide and its systemic effects, treatment typically includes the provision of active clotting factors via transfusion therapy and exogenous vitamin K administration (11). Seventy-seven percent of dogs received a transfusion. The location of hemorrhage did not correlate with the type of or need for a transfusion. However, patients whose treatment included a transfusion did significantly increase the rate of survival to discharge. When assessing the survival rate of dogs who received a transfusion vs. those who did not, 36% of dogs that did not receive a transfusion died prior to discharge or were euthanized during hospitalization. One dog who did not receive a transfusion was discharged to be euthanized by its primary veterinarian, making the total percentage of dogs that did not survive or receive a transfusion 43%. This is in contrast to 4% of dogs who did receive a transfusion not surviving to discharge.

In this study, the prognosis for patients who presented for anticoagulant rodenticide toxicity and clinical hemorrhage was excellent with 87% patients surviving to discharge, which is similar to previous reported survival of 83% (5). The location of hemorrhage and number of sites hemorrhaging did not correlate with survival to discharge. Of the patients that did not survive four dogs were euthanized and four died. Of the four that were euthanized, three were euthanized due to financial concerns while one dog was euthanized due to guarded prognosis secondary to the severity of pulmonary compromise and pulmonary hemorrhage.

Limitations of our study include the retrospective nature of the study design, which did not allow for standardization in diagnosis or treatment of patients with suspected anticoagulant rodenticide toxicities. Additionally, the inclusion of patients only with known history of ingestion or patients whose owners chose to have gas chromatography mass spectroscopy testing run is a limiting factor as it may have excluded patients from the study leaving a small number of patients included in this study. While reasons for euthanasia were evaluated based on retrospective review of records the nature of this study makes full elucidation of outcome difficult.

## Conclusions

In conclusion, this study highlights that dogs with anticoagulant rodenticide intoxication are likely to present with diverse locations of hemorrhage, including cutaneous and mucosal hemorrhage. While the conventional cavitary hemorrhage associated with anticoagulant rodenticide intoxication is noted, it is pertinent that anticoagulant rodenticide toxicity remains on the differential list in patients presenting with cutaneous or mucosal hemorrhage including hemorrhage from the oral cavity, nasal cavity, eyes, gastrointestinal tract or urinary tract.

## Data Availability Statement

The data analyzed in this study is subject to the following licenses/restrictions: Medical records were retrospectively reviewed. Requests to access these datasets should be directed to rwalton@iastate.edu.

## Ethics Statement

Ethical review and approval was not required for the animal study because this was a retrospective study. Written informed consent for participation was not obtained from the owners because this was a retrospective study.

## Author Contributions

RW contributed to research idea, data collection, and manuscript writing. SS and BE contributed to data collection and manuscript writing. JM and LY contributed to statistical analysis and manuscript writing. All authors contributed to the article and approved the submitted version.

## Conflict of Interest

The authors declare that the research was conducted in the absence of any commercial or financial relationships that could be construed as a potential conflict of interest.

## Publisher's Note

All claims expressed in this article are solely those of the authors and do not necessarily represent those of their affiliated organizations, or those of the publisher, the editors and the reviewers. Any product that may be evaluated in this article, or claim that may be made by its manufacturer, is not guaranteed or endorsed by the publisher.
